# In Silico Analysis Identified Putative Pathogenic Missense Single Nucleotide Polymorphisms (SNPs) in the Human *HNF1A* Gene

**DOI:** 10.3390/ijms26083768

**Published:** 2025-04-16

**Authors:** Hitham Aldharee, Hamdan Z. Hamdan

**Affiliations:** 1 Department of Pathology, College of Medicine, Qassim University, Buraidah 51452, Saudi Arabia; h.abualbasher@qu.edu.sa; 2 Unit of Genetic Diabetes, Abdullah Al Othaim Diabetes Center, Medical City, Qassim University, Buraidah 51452, Saudi Arabia

**Keywords:** MODY, MODY-3, HNF1A, diabetes, genetics, rare disorders

## Abstract

Maturity-onset diabetes of the young (MODY) is a rare genetic condition that affects children, adolescents, and adults. Studies have shown that genetic changes in the *HNF1A* gene are associated with MODY-3. However, most of the causative variants and the molecular mechanisms remain underexplored. This study aims to better understand MODY-3 by investigating HNF1A-missense variants with clinical uncertainty. Various bioinformatics tools were utilised to address the clinical uncertainty of missense variants in the *HNF1A* gene that have not been linked with HNF1A-related conditions, sourced from the Genome Aggregation Database (GnomAD v4.1.0). Among the clinically uncertain 2444 variants, only 138 were classified as missense with clinically uncertain significance. Results show that four variants (Arg168Cys, Glu275Ala, Gly375Asp and Val411Phe) were consistently predicted as pathogenic by all tools. The allele frequency (AF) of the commonly predicted disease-causing variants was very low in the global population. The assessment of the secondary structure of filtered variants indicates that variants (Arg168Cys and Glu275Ala) are located in the helical region of the HNF1A protein. At the same time (Gly375Asp and Val411Phe) are found in the protein’s coil, suggesting structural changes at the site of variations. The prediction of protein stability was conducted using I-Mutant and MuPro. Both tools collectively indicate decreased protein stability for the variants (Arg168Cys, Glu275Ala, Gly375Asp and Val411Phe). Predicting the protein’s 3D structure for the HNF1A wild-type and mutants indicates potential structural damages in Arg168Cys and Gly375Asp. Additionally, results show that the amino acids at the variation sites of the variants (Arg168Cys, Glu275Ala, Gly375Asp and Val411Phe) were highly conserved. To conclude, 4 out of the 138 missense variants labelled as uncertain significance were found to be consistently pathogenic using in silico tools in this study. Our findings aim to support variant interpretation, understand the genotype–phenotype association of diabetes, and provide better healthcare services for patients with diabetes.

## 1. Introduction

Maturity-onset diabetes of the young (MODY; MIM #606391) is a rare form of genetic diabetes that was first described in 1974 [1,2]. MODY comprises hyperglycaemia in young people (under 25 years of age) caused by monogenic alterations of genes responsible for beta-cell function and insulin action, representing 1–5% of diabetic cases [2,3,4]. It is categorised into subtypes (MODY1-14), each linked with a defect in a specific gene and mostly inherited in an autosomal dominant pattern [1,5]. MODY-3 is the most prevalent subtype, caused by variants in the hepatocyte nuclear factor 1-alpha gene (*HNF1A*; OMIM #142410), which is responsible for producing the HNF1A protein that plays crucial roles in regulating the activity of genes involved in blood glucose level control.

The *HNF1A* is mapped to (12q24.31) and comprises 10 exons, which encode 631 amino acids and the HNF1A protein [5,6,7].

According to published data by the National Human Genome Research Institute (NHGRI), an individual genome is estimated to contain thousands of single nucleotide polymorphisms (SNPs), insertion/deletion, and structural variants. These variations may be pathogenic, linked to some abnormal conditions, present benign effects, or are uncertain. Regarding MODY-3, a list of variants have been identified within the HNF1A gene: missense, Indels, frameshift, inframe deletion, inframe insertion, and splice sites [5,8]. Although most genetic changes are characterised as SNPs, whether the reported variants alter the normal expression and function of the HNF1A protein is still under-studied. Furthermore, missense variants are known for their effects on the translated messenger RNA (mRNA) and expressed protein. According to the Genome Aggregation Database (GnomAD v4.1.0), hundreds of missense variants have been reported in the HNF1A gene; however, some are classified as pathogenic, likely pathogenic, or benign, while the remaining variants are under investigation and classified as uncertain in terms of their clinical effects.

Identifying pathogenic SNPs involved in the causation of rare diseases such as MODY is complex and challenging due to the requirement of many cases, multiple genetic testing, and SNPs prioritisation. An alternative, efficient, and reliable approach is recommended to support the discrimination of unclassified variants and determine whether they are causative and affect the disease’s prognosis. Clinically, computational-based evidence is approved as a criterion for classifying variants by the American College of Medicine (ACMG) and the Association of Molecular Pathology (ACP) [9]. Moreover, it has been reported that most algorithms used for missense variants show an accuracy of 65–80% in examining disease-causative variants [10]. Therefore, in silico analysis through bioinformatics tools could prove to be a promising strategy to address this limitation in MODY, given the capabilities of such tools in differentiating disease-causative from non-causative variants.

This study aims to enhance the genetic understanding of MODY-3 by detailing the potential effects of missense variants with uncertain clinical significance reported by the GnomAD v4.1.0 database on the normal expression and function of the HNF1A protein.

## 2. Results

### 2.1. Variant Recruitment and Selection

Searching the GnomAD v4.1.0 databases revealed 2442 variants in the HNF1A gene. Among these, 817 were missense variants, with the remaining distributed across splice site, 5′ UTR, 3′ UTR, frameshift, in-frame insertion and deletion, intronic, start-lost, stop-gained, stop-retained, and synonymous variants. We selected a total of 138 missense variants, which cause changes in amino acids and are categorised as uncertain significance (US), for further bioinformatic analysis.

### 2.2. Predicting Pathogenicity of Variants

The selected missense–US variants were analysed using the following bioinformatics tools to assess their potential pathogenicity: SNPs&GO, Meta-SNP, Polyphen2, PANTHER, SIFT, PhD-SNP, and SNAP2. The results indicate that all tools collectively predicted only four out of 138 variants as pathogenic (Table 1). Individual reports show that SNPs&GO predicted 135 variants as disease-causing, Meta-SNP identified 24 as disease-causing, Polyphen2 indicated 86 as probably damaging, PANTHER found 40 as disease-causing, SIFT identified 46 as disease-causing, PhD-SNP reported 22 as disease-causing, and SNAP2 indicated 79 as disease-causing. Ultimately, the variants commonly predicted to be pathogenic were selected for further analysis.

Further analysis of the commonly predicted variants using AlphaMissense (https://www.science.org/doi/10.1126/science.adg7492, accessed on 24 January 2025), an AI-based tool, yielded the following scores: p.Arg168Cys (0.97, Pathogenic), p.Glu275Ala (0.85, Pathogenic), p.Gly375Asp (0.92, Pathogenic), and p.Val411Ala (0.78, likely pathogenic). These high-confidence predictions support our earlier findings and reinforce the deleterious nature of these missense mutations.

### 2.3. Determining Variant Frequency

The frequency of the commonly predicted disease-causing variants was assessed using dbSNP (https://www.ncbi.nlm.nih.gov/snp/, accessed on 24 January 2025). The results indicate that the frequency of variant I among the global population was (0.00006), variant II (0.00004), variant III (0.00004), and variant IV (0.00004). The frequency of all variants is very low, suggesting a high probability of pathogenicity.

### 2.4. Predicting Protein Secondary Structure

Quick2D, a comprehensive tool, was used to predict secondary structures. The results indicate that Arg168Cys and Val411Phe reside within the alpha helix region, which may disrupt protein integrity and stability. Conversely, Glu275Ala is located in the coil region, while Gly375Asp is located in the strand region, which could influence beta-sheet formation and protein–protein interaction.

### 2.5. Predicting Protein Stability

The stability of proteins associated with common pathogenic variants was evaluated using the MUpro and I-Mutant bioinformatics tools. The results indicated that variants I, II, III, and IV were typically predicted to reduce protein stability, suggesting a loss of function in the HNF1A protein (Table 2).

### 2.6. Protein–Protein Interaction

**Figure 1:**
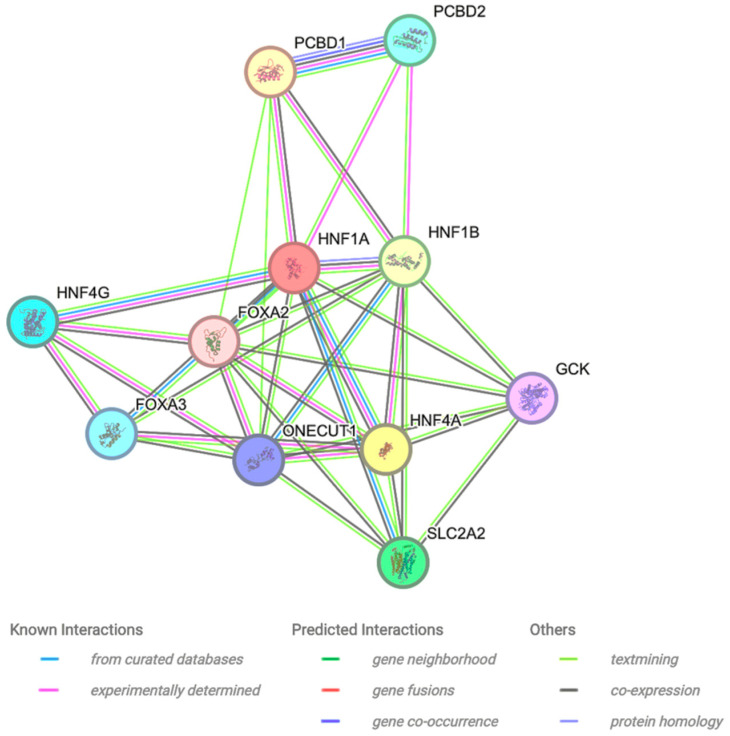
Protein–protein interaction using the STRING bioinformatic tool. The interaction prediction was based on known interactions, predicted interaction, and other data such as textmining, co-expression, and protein homology.

The interactor proteins of HNF1A were examined using the STRING bioinformatics tool. The findings indicate that the HNF1A protein interacts with PCBD1, PCBD2, HNF1B, HNF4G, FOXA2, FOXA3, GCK, HNF4A, ONECUT1, and SLC2A2 (Figure 1). Among these, PCBD1 displays the closest interaction, directly interacting with HNF1A.

### 2.7. Predicting Protein 3D Structure

**Figure 2:**
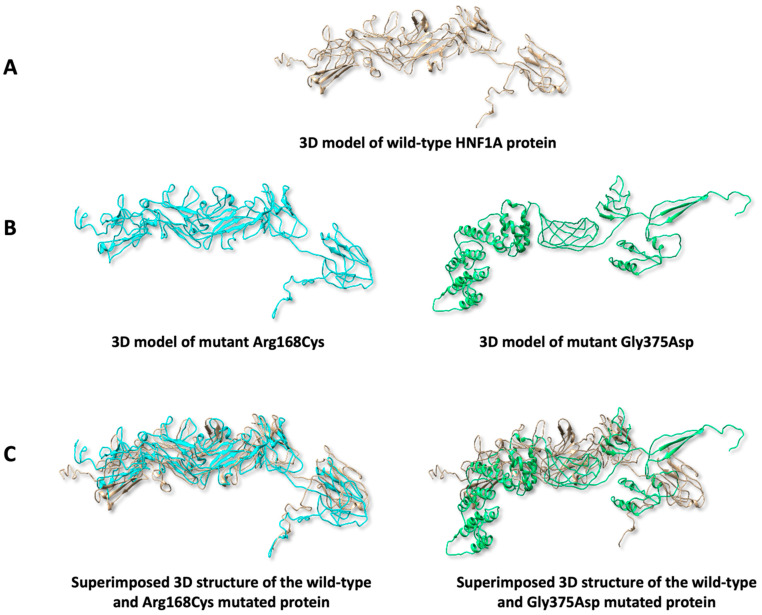
The three-structure (3D) analysis of the wild-type HNF1A protein and mutants. (**A**) The 3D model of the normal HNF1Aprotein. (**B**) The 3D model of the mutants (Arg168Cys and Gly375Asp). (**C**) Superimposed models of the mutants with the wild-type HNF1A protein.

The 3D structural analysis of the HNF1A wild-type protein and the predicted pathogenic variants was carried out using I-TASSER and UCSF Chimera tools. Results indicate that structural damage was observed in Arg168Cys (in terms of replacing buried charged residue R with a non-charged residue C, with RSA = 0.4%) and Gly375Asp (manifested as introducing a charged residue in a place of uncharged residue with RSA = 1.1%) (Figure 2B), while no damage was seen in the structure of the other mutants. Additionally, the 3D models of the mutants were superimposed onto the wild-type model for manual comparison of the structural similarities. Findings suggest that the variant Arg168Cys shows the highest similarity to the wild-type protein compared to Gly375Asp (Figure 2C).

### 2.8. Conservation of the HNF1A Amino Acids

**Figure 3:**
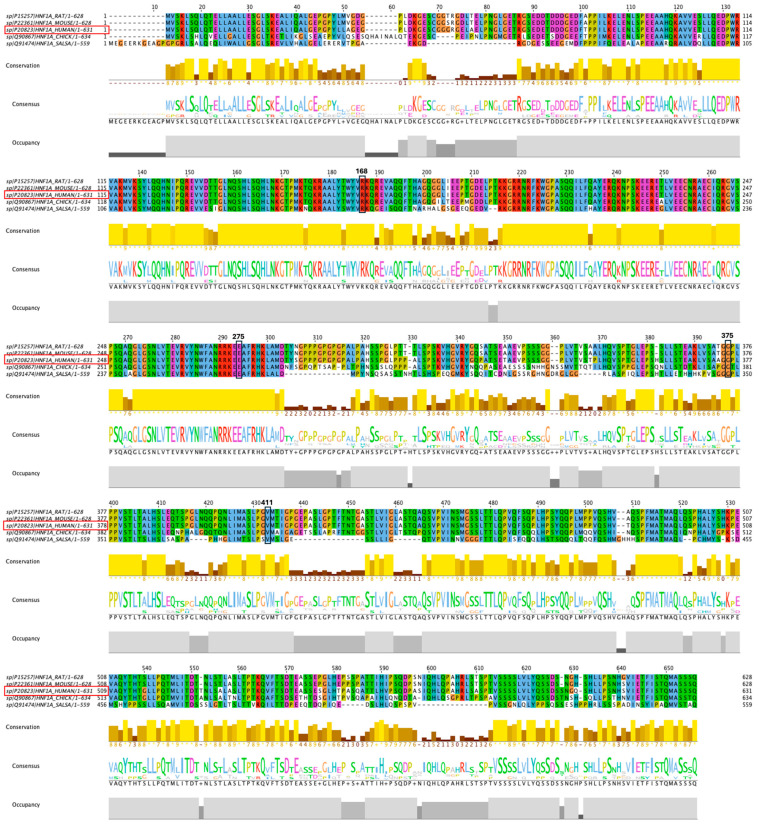
Conservation analysis of amino acids of the HNF1A protein using Jalview software. Variants of interest are highlighted in black boxes. Red boxes indicate the amino acid sequence of the human *HNF1A*.

Results from multiple sequence alignments using the Jalview software reveal that all amino acids at the variation sites of variants I, II, III, and IV are highly conserved across all selected species (Figure 3). These findings suggest a functional and/or evolutionary significance of the residues at the variation sites.

## 3. Materials and Methods

### 3.1. Variants Recruitment and Selection

Missense variants of the HNF1A gene were retrieved from the GnomAD v4.1.0 database (https://gnomad.broadinstitute.org, accessed on 3 October 2024). A file listing all the variants of the HNF1A gene was manually filtered using an MS Excel-supported file. Only missense variants classified as variants of unknown significance (VUS), have not been reported in ClinVar with HNF1A- related conditions, and located within the coding region of the HNF1A gene were selected for further analysis. The transcript (ENST00000257555.11) was used as a reference in accordance with the Human Genome Variation Society (HGVS) guidelines. The selected variants were analysed using various bioinformatics tools for variant prioritisation.

### 3.2. Predicting Pathogenicity of Variants

The following bioinformatics tools were used to predict and distinguish pathogenic variants from non-pathogenic ones: SNPs&GO (https://snps.biofold.org/snps-and-go/snps-and-go.html, accessed on 12 October 2024), Meta-SNP (https://snps.biofold.org/meta-snp/, accessed on 26 October 2024), Polymorphism Phenotyping v2 (Polyphen2) (http://genetics.bwh.harvard.edu/pph2/, accessed on 13 November 2024), protein analysis through evolutionary relationships (PANTHER) (https://pantherdb.org, accessed on 21 November 2024), sorting intolerant from tolerant (SIFT) (https://sift.bii.a-star.edu.sg, accessed on 5 December 2024), predictor of human deleterious SNP (PhD-SNP) (https://snps.biofold.org/phd-snp/phd-snp.html, accessed on 17 December 2024), and SNAP2 (https://bio.tools/snap2, accessed on 26 December 2024), and AlphaMissense (https://www.science.org/doi/10.1126/science.adg7492, accessed on 24 January 2025) [11].

### 3.3. Determining Variant Frequency

The Single Nucleotide Polymorphism Database (dbSNP) was used to calculate the variation allele frequency (VAF) of selected variants predicted as pathogenic by all the bioinformatic tools (https://www.ncbi.nlm.nih.gov/snp/, accessed on 2 January 2025).

### 3.4. Predicting Protein Secondary Structure

The prediction of the secondary structure of the HNF1A natural protein and the proteins of selected variants predicted as pathogenic by all the bioinformatics tools was conducted using the Quick2D (https://toolkit.tuebingen.mpg.de/tools/quick2d, accessed on 23 March 2025).

### 3.5. Predicting Protein Stability

The protein stability (PS) of selected variants, which were predicted as pathogenic by all the bioinformatic tools, was assessed using MUpro (http://mupro.proteomics.ics.uci.edu, accessed on 15 January 2025) and I-Mutant 2.0 (https://folding.biofold.org/i-mutant/i-mutant2.0.html, accessed on 18 January 2025).

### 3.6. Protein–Protein Interaction

The assessment of HNF1A interactive proteins was conducted using the Search Tool for the Retrieval of Interacting Genes/Proteins (STRING) (https://string-db.org, accessed on 18 January 2025), which relies on co-expression, experimental data, function, and gene fusion and interaction.

### 3.7. Predicting Protein 3D Structure

The 3D structure of the HNF1A natural protein and the proteins of selected variants, predicted to be pathogenic by all bioinformatic tools, were predicted using the Iterative Threading ASSEmbly Refinement (I-TASSER) tool (https://seq2fun.dcmb.med.umich.edu//I-TASSER/, accessed on 21 January 2025). Additionally, the Missense3D tool (https://missense3d.bc.ic.ac.uk/missense3d/, accessed on 24 January 2025), was employed to predict any possible structural damage in the 3D protein structure, caused by the amino acid substitutions. This tool calculates the relative solvent accessibility (RSA%) of the protein following the introduction of the substituted amino acid. It provides both quantitative and qualitative assessments of the structural impact. The UCSF Chimera software (https://www.cgl.ucsf.edu/chimera/, accessed on 24 January 2025) was used to visualise the 3D structure of the proteins and to overlay the normal and mutated proteins for comparative analysis of structural variations.

### 3.8. Conservation Analysis

The conservation assessment of the HNF1A protein among the species was conducted using Jalview software. The amino acid sequence of the human HNF1A protein, along with those of other species, was retrieved from the NCBI’s HomoloGene sub-database (https://www.ncbi.nlm.nih.gov, accessed on 25 January 2025). Multiple sequence alignment (MSA) conservation analysis, and consensus were carried out on Jalview software to examine the conserved amino acids among selected species and visualised by using Jalview software (version 2.11.4.1).

## 4. Discussion

The in silico approach is widely used and enables researchers to conduct targeted and functional variant analyses for specific diseases. In this study, we investigate missense variants in the *HNF1A* gene that are classified as having uncertain significance and have not been linked with HNF1A- related conditions. HNF1A is a well-known transcription factor, and its variants are strongly associated with the development of maturity-onset diabetes of the young (MMODY).

Expression analysis has shown that HNF1A, as a transcription factor, regulates key pathways involved in glucose metabolism, such as glycolysis, the Krebs cycle, and the respiratory chain. These pathways constitute a cascade system that directly contributes to insulin secretion, termed glucose-stimulated insulin secretion [12]. Additionally, HNF1A activates other transcription factors that also affect glucose metabolism [13,14]. Consequently, variants in the HNF1A gene are clinically linked to the pathogenesis of MODY.

The initial analysis identified 138 missense variants with clinically uncertain significance. The study’s major finding was the identification of four missense SNPs (Cys168Arg, Glu275Ala, Gly375Asp and Val411Phe) predicted to be pathogenic using seven bioinformatics tools. The variants (Arg168Cys, Glu275Ala, Gly375Asp and Val411Phe) were predicted to decrease protein stability. Additionally, three-dimensional (3D) analysis predicts that the variants (Arg168Cys and Gly375Asp) are associated with 3D structural damage.

The HNF1A protein is a transcription factor that possesses the typical structure of transcription factors, comprising an N-terminal dimerization domain, a DNA-binding domain, and a C-terminal transactivation domain [14,15,16]. The DNA-binding domain plays a crucial role in identifying the promoter region of target genes, facilitating the assembly and initiation of transcription machinery [15]. Therefore, structural and functional alterations in this sensitive region may provide a rationale for the abnormalities observed in MODY3 cases. Variant I (Arg168Cys) also lies within the α-helix of the DNA-binding domain. The substitution of arginine—a large, positively charged amino acid—for cysteine, a smaller, polar, and non-charged amino acid, may disrupt DNA binding with HNFA1. Although both amino acids are hydrophilic, arginine is structurally the largest amino acid in this context and carries a positive charge, whereas cysteine is structurally smaller and uncharged. Also, introducing a cysteine residue may also confer redox sensitivity. Under oxidative conditions, cysteine could form disulfide bonds that alter protein folding or promote dimerization, potentially affecting nuclear localisation, stability, or protein–protein interaction [17]. These biophysical changes support the structural and functional disruption predicted by the current bioinformatics analysis.

In addition, variant VI (Gly375Asp) is predicted to cause structural damage and affect HNF1A activity functionally. This variant is found in the C-terminal transactivation domain. While it is not directly involved in DNA binding, its role influences the transcription rate of target genes [18]. Amino acid substitutions may alter the interaction between HNF1A and its activators or co-activators, ultimately resulting in reduced transcriptional activity. Also, this region is classified as an intrinsically disordered area, enabling interactions with other transcription factors, repressors, and activators that regulate transcription activity.

From an evolutionary perspective, all the investigated variant positions are highly conserved among vertebrates, highlighting their critical role in protein function across species [19]. The high degree of conservation in this transcription factor may be attributed to its interactions with multiple proteins across various species. Therefore, these conserved regions are thought to resist the negative selection pressure associated with their variants [20].

Post-translational modifications (PTMs) of HNF1A, including phosphorylation and acetylation, are recognised as regulators of its transcriptional activity. Although the identified variants are not located within known PTM sites, it remains possible that structural changes induced by these mutations could affect the accessibility or recognition of nearby PTM sites [21].

Previous studies on MODY3 have indicated that the location of variants within the HNF1A gene can influence the disease phenotype [22,23]. Specifically, variants in the DNA-binding domain are linked to reduced HNF1A-DNA binding, resulting in decreased transcriptional activity of target genes. Moreover, certain variants may affect nuclear localisation [24]. Consequently, variants in the DNA-binding domain are typically regarded as more severe than those in the transactivation domain. Clinically, this severity presents as an earlier age of onset and severe hyperglycaemia, often necessitating early insulin therapy [22,23].

Additionally, the epigenetic regulation of HNF1A expression and its target genes may affect phenotypic variability, while lifestyle factors such as diet and metabolic stress can further alter disease severity. Thus, they can influence disease onset and severity even among individuals carrying the same pathogenic variant [25].

## 5. Conclusions

To date, the variants investigated have been classified as variants of uncertain significance (VUS). However, based on the current computational analysis, they may warrant reclassification as likely pathogenic. Nonetheless, this study has limitations that need to be addressed. Firstly, although the reported alleles are rare, destabilise the HNF1A protein, and are predicted to be pathogenic, they have not yet been associated with MODY onset cases. This could be attributed to the fact that MODY cases are rare, with most either undiagnosed or misdiagnosed. Additional, molecular dynamics simulation and in vitro functional assays of the mutant proteins, such as differential scanning fluorimetry and circular dichroism spectroscopy, would provide practical validation of the variants’ pathogenicity and their impact on protein function and stability.

## Figures and Tables

**Table 1:** List of variants commonly predicted to be pathogenic by all tools.

					Polyphen2		SNPs&Go		MetaSNP		SIFT		Panther		PHD SNP		SNAP2		AlphaMissense	
S.No	Chr:bp	Alleles	AA	AA Coord	Pred	Prob	Pred	Prob	Pred	Score	Pred	Score	Pred	Preservation Time	Pred	Score	Pred	Score	Pre	Score
I	120989008	C/T	Cys/Arg	168	ProD	1	D	0.994	D	0.56	D	0.03	D	0.693	D	0.678	D	0.625	P	0.97
II	120994274	A/C	Glu/Ala	275	ProD	1	D	0.989	D	0.502	D	0.01	D	0.729	D	0.562	D	0.695	P	0.85
III	120996557	G/T	Gly/Asp	375	ProD	0.998	D	0.992	D	0.728	D	0	D	0.698	D	0.68	D	0.745	P	0.92
IV	120996664	G/T	Val/Phe	411	ProD	1	D	0.993	D	0.669	D	0.02	D	0.602	D	0.609	D	0.69	LP	0.78

Key: Chr: Chromosome; bp: base pair; AA: amino acid; AA coord: amino acid coordinate; Pred: prediction; Prob: probability; D: damaging; P: pathogenic; LP: likely pathogenic.

**Table 2:** List of variants showing decreases in the protein stability.

				I-Mutant		MuPro	
Variant No.	rs ID	AA	AA Coord	Stability	RI	Stability	Score
I	rs764434453	Cys/Arg	168	decrease	3	decrease	−0.57
II	rs199890776	Glu/Ala	275	decrease	2	decrease	−0.37
III	rs1315462017	Gly/Asp	375	decrease	5	decrease	−0.24
IV	rs767284188	Val/Phe	411	decrease	9	decrease	−1.43

## Data Availability

All relevant data are within the manuscript.
